# Decentralized Primal-Dual Proximal Operator Algorithm for Constrained Nonsmooth Composite Optimization Problems over Networks

**DOI:** 10.3390/e24091278

**Published:** 2022-09-11

**Authors:** Liping Feng, Liang Ran, Guoyang Meng, Jialong Tang, Wentao Ding, Huaqing Li

**Affiliations:** 1 Department of Computer Science, Xinzhou Teachers University, Xinzhou 034000, China; 2 Chongqing Key Laboratory of Nonlinear Circuits and Intelligent Information Processing, College of Electronic and Information Engineering, Southwest University, Chongqing 400715, China; 3 Department of Mathematics, Xinzhou Teachers University, Xinzhou 034000, China

**Keywords:** nonsmooth optimization, decentralized optimization, primal-dual algorithm, uncoordinated stepsizes, distributed signal processing, information processing

## Abstract

In this paper, we focus on the nonsmooth composite optimization problems over networks, which consist of a smooth term and a nonsmooth term. Both equality constraints and box constraints for the decision variables are also considered. Based on the multi-agent networks, the objective problems are split into a series of agents on which the problems can be solved in a decentralized manner. By establishing the Lagrange function of the problems, the first-order optimal condition is obtained in the primal-dual domain. Then, we propose a decentralized algorithm with the proximal operators. The proposed algorithm has uncoordinated stepsizes with respect to agents or edges, where no global parameters are involved. By constructing the compact form of the algorithm with operators, we complete the convergence analysis with the fixed-point theory. With the constrained quadratic programming problem, simulations verify the effectiveness of the proposed algorithm.

## 1. Introduction

Recently, the distributed data processing methods based on multi-agent networks have received much attention. The traditional methods put all the data into one machine and perform the computation centrally. However, as the size of data continues to grow, this kind of centralized strategy is limited by the computing power of the hardware. In contrast to this, the distributed methods distribute computing tasks to agents over decentralized networks [1,2]. Each agent keeps an arithmetic unit and a memory unit. The agents interact with each other through communication links, and this communication occurs only among the neighboring agents. Under these conditions, the distributed methods can effectively solve the optimization problems common to sensor networks [3], economic dispatch [4,5,6], machine learning [7,8] and dynamic control [9].

The existing decentralized algorithms have included some successful results [10,11,12,13,14,15]. Previous works considered the problem models composed of a single function. With a fixed stepsize, Shi et al. designed EXTRA [10], which can exactly converge to the optimal solution. Lei et al. studied problems with bound constraints and proposed the primal-dual algorithm [11]. In addition, recent works [16,17,18] investigated a general distributed optimization with an objective function by designing decentralized subgradient-based algorithms, but diminishing or non-summable step-sizes are utilized, which may cause slow convergence rates [19].

In order to make full use of these special properties, some scholars have studied the nonsmooth composite optimization problems, which possess smooth and nonsmooth structures. By extending EXTRA to the nonsmooth combinational optimization, Shi et al. proposed PG-EXTRA [20]. Li et al. introduced the network-independent stepsize to PG-EXTRA and then developed NIDS [21]. In addition, Aybat et al. proposed DPDA-D [22] for time-varying networks. Considering the situation that the nonsmooth term cannot be split, Xu et al. proposed the [23]. PG-ADMM [24] was designed based on the distributed alternating direction multiplier method. Particularly, the nonsmooth combinational optimization problems also include a class of problems consisting of three functions and a linear operator. This structure is mainly discussed in the centralized optimization [25,26,27,28], and recently some distributed works also appear in [29,30]. In this paper, inspired by the constrained optimization problem [31], we study the constrained nonsmooth composite optimization problems over networks.

The contributions of this paper can be summarized as follows:

1. This paper focuses on an optimization problem with partially smooth and nonsmooth objective functions, where the decision variable satisfies local equality and feasible constraints, unlike these works [10,16,18,19,20,21] without considering any constraints. Then, to solve this problem, we propose a novel decentralized algorithm by combining primal-dual frame with the proximal operators, which avoids the estimation of subgradients for nonsmooth terms.
2. Different from existing node-based methods [16,17,18,19,20,21], the proposed algorithm adopts an edge-based communication pattern that explicitly highlights the process of information exchange among neighboring agents and further gets rid of the dependence on Laplacians [13]. Such a consideration also makes it possible to use uncoordinated stepsizes instead of commonly global or dynamic ones [10,12,16,18,19,21].
3. By employing the first-order optimal conditions and fixed-point theory of operators, the convergence is proved, and its sublinear rate $O\left(1/k\right)$ (*k* is the number of iteration); i.e., at most, $O\left(1/\epsilon \right)$ iterations in order to reach an accuracy of ϵ is established.

*Organization:* The rest of this paper is organized as follows. In Section 2, the necessary notations and basic knowledge are first provided, and then we describe the optimization problem over the networks and necessary assumptions. Section 3 supplies the development of the proposed decentralized algorithm. In Section 4, the convergence analysis for the proposed algorithm is provided. In Section 5, we use the simulation experiments to verify the theoretical analysis. Finally, conclusions are given in Section 6.

## 2. Preliminaries

In this section, we introduce the notations involved in this paper. Meanwhile, the objective problem and its explanation are also supplied.

### 2.1. Graph Theory and Notations

The knowledge of graph theory is used to construct the mathematical model of the communication network. Let $G=\left(V,E\right)$ describe the network as a graph, where V is the set of vertices and E⊂V×V is the set of edges. For an agent i∈V, $N_{i}$ denotes the set of its neighbors. Let the unordered pair (i,j)∈E represent the edge between agent *i* and agent *j*. However, (i,j) or (j,i) is still order, i.e., the variables with respect to them are different.

Next, we explain the notations that appear in this paper. Let R represent the set of real numbers. Therefore, $R^{n}$ denotes the *n*-dimensional vector space, and $R^{n\times m}$ denotes the set of all *n*-row and *m*-column real matrices. We define $I_{n}$ as the *n*-dimensional identity operator, $0_{n}$ as the *n*-dimensional null vector, and $0_{n\times n}$ as the null matrix. If their dimensions are clear from the context, we omit their subscript. Then, blkdiag{P,Q} is the block diagonal matrix grouped by matrices *P* and *Q*. For a matrix *P*, let *M* be its transpose. We denote ${∥x∥}_{P}=\sqrt{x^{⊤}Px}$ as the induced norm with matrix *P*. The subdifferential of function *f* is ∂f, where $\partial f\left(x\right)=\left\{p|\forall q\in R^{n},f\left(x\right)+p^{⊤}\left(q-x\right)\leq f\left(q\right)\right\}$. The conjugate function $f^{*}$ is defined by $f^{*}\left(p\right)=\sup_{q\in R^{n}}\left\{\langle p,q\rangle -f\left(q\right)\right\}$. For a positive constant μ, the resolvent of the proximal operator is $\mathrm{pro}x_{\mu f}\left(y\right)={\left(I+\mu \partial f\right)}^{-1}\left(y\right)=\arg\min_{x}f\left(x\right)+\frac{1}{2\mu }{∥x-y∥}^{2}$, while the resolvent with respect to the matrix *M* is ${\mathrm{prox}}_{f}^{M}\left(y\right)={\left(I+M^{-1}\partial f\right)}^{-1}\left(y\right)=\arg\min_{x}f\left(x\right)+\frac{1}{2}{∥x-y∥}_{M}^{2}$. Moreover, let S represent the optimal solution set of a solvable optimization problem over networks.

### 2.2. Decentralized Optimization Problem

The constrained composite optimization problem over networks studied in this paper is based on the network G={V,E} with *m* agents. Specifically, the formulation of the problem is established as follows:

(1a) $$\begin{array}{cc} \; & \min_{\tilde{x}\in R^{n}}\;\;\sum_{i=1}^{m}f_{i}\left(\tilde{x}\right)+g_{i}\left(\tilde{x}\right), \end{array}$$

(1b) $$\begin{array}{cc} \; & \;s.t.\;\;\;A_{i}\tilde{x}=b_{i},\;i=1,\cdots ,m, \end{array}$$

(1c) $$\begin{array}{cc} \; & \;\;\;\;\;\;\;\;\;\;\tilde{x}\in {⋂}_{i=1}^{m}\Omega_{i}. \end{array}$$

In problem (1), $\tilde{x}\in R^{n}$ is the decision variable; $f_{i}:R^{n}\to R\cup \left\{+\infty \right\}$ and $g_{i}:R^{n}\to R\cup \left\{+\infty \right\}$ are two private cost functions to agent *i*, where the former has the Lipschitz continuous gradient, but the latter may be nonsmooth; $b_{i}\in R^{r}$ is a vector and $A_{i}:R^{n}\to R^{r}$ is a linear operator. Convex set $\Omega_{i}$ gives the box constraints to the decision variable of agent *i*.

To clarify the properties of problem (1), the following necessary assumption is given.

**Assumption** **1.** *For any agent i∈V:*

*(i)*  *The cost function $f_{i}$ is Lipschitz continuous and convex; i.e., if we consider the positive Lipschitz constant $\beta_{i}$, then it holds the inequality for the gradient $\nabla f_{i}$:*
(2) $$\begin{array}{c} {∥\nabla f_{i}\left(\tilde{x}\right)-\nabla f_{i}\left(\tilde{y}\right)∥}^{2}\leq \beta_{i}{\left(\tilde{x}-\tilde{y}\right)}^{⊤}\left(\nabla f_{i}\left(\tilde{x}\right)-\nabla f\left(\tilde{y}\right)\right). \end{array}$$
*(ii)*  *The local cost function $g_{i}$ is a nonsmooth and convex function.*
*(iii)*  The optimal solution ${\tilde{x}}^{*}$ to objective problem (1) exists, which satisfies both the equality constraints and the box constraints.
*(iv)*  *The graph G is undirected and connected.*

Note that the cost functions $f_{i}$ and $g_{i}$ are separable. Hence, we introduce the consensus constraint to transform problem (1) into the structure that can be computed in a decentralized manner:

(3a) $$\begin{array}{cc} \; & \min_{x_{1},\cdots ,x_{m}}\sum_{i=1}^{m}f_{i}\left(x_{i}\right)+g_{i}\left(x_{i}\right), \end{array}$$

(3b) $$\begin{array}{cc} \; & \;\;\;\;s.t.\;\;\;\;A_{i}x_{i}=b_{i},\;i=1,\cdots ,m, \end{array}$$

(3c) $$\begin{array}{cc} \; & \;\;\;\;\;\;\;\;\;\;\;\;\;x_{i}=x_{j},\;i=1,\cdots ,m,\;j\in N_{i}, \end{array}$$

(3d) $$\begin{array}{cc} \; & \;\;\;\;\;\;\;\;\;\;\;\;\;x_{i}\in \Omega_{i}. \end{array}$$

Define the set $A_{i}=\left\{z\in R^{n}\left|A_{i}z=b_{i}\right.\right\}$ and consider the indicator function

$$\begin{array}{c} \delta_{C}\left(e\right)=\left\{\begin{array}{c} 0,\;\;\;\;\;\;\;\;\;\;e\in C,\\ +\infty ,\;\;\;\;\;e\notin C, \end{array}\right. \end{array}$$

such that Problem (3) can be processed by the penalty function method. For i∈V and $j\in N_{i}$, let $C_{ij}=I$ if i<j and $C_{ij}=-I$ otherwise. Thus, Problem (3) is equivalent to the following problem:

(4a) $$\begin{array}{cc} \; & \min_{x_{1},\cdots ,x_{m}}\sum_{i=1}^{m}f_{i}\left(x_{i}\right)+g_{i}\left(x_{i}\right)+\delta_{A_{i}}\left(x_{i}\right)+\delta_{\Omega_{i}}\left(x_{i}\right), \end{array}$$

(4b) $$\begin{array}{cc} \; & \;\;\;\;s.t.\;\;\;\;C_{ij}x_{i}+C_{ji}x_{j}=0,\;i=1,\cdots ,m,\;j\in N_{i}. \end{array}$$

Then, let $x=col\left(x_{1},\ldots ,x_{m}\right)$ be the global variable. For i∈V and $j\in N_{i}$, we introduce a linear operator $N_{\left(i,j\right)}:x\mapsto {\left({\left(C_{ij}x_{i}\right)}^{⊤},{\left(C_{ji}x_{j}\right)}^{⊤}\right)}^{⊤}$, which generates the edge-based variable from *x*. With the set $C_{\left(i,j\right)}=\left\{{\left(z_{1}^{⊤},z_{2}^{⊤}\right)}^{⊤}\left|z_{1}+z_{2}=0\right.\right\}$, the constraint in the problem (4) can be transformed into another penalty function. Therefore, the problem (1) is finally equivalent to the following problem:

(5) $$\begin{array}{cc} \min_{x_{1},\cdots ,x_{m}} & \sum_{i=1}^{m}f_{i}\left(x_{i}\right)+g_{i}\left(x_{i}\right)+\delta_{A_{i}}\left(x_{i}\right)+\delta_{\Omega_{i}}\left(x_{i}\right)+\sum_{i=1}^{m}\sum_{\left(i,j\right)\in E}\delta_{C_{\left(i,j\right)}}\left(N_{\left(i,j\right)}x\right). \end{array}$$

Based on the problem (5), we design a novel decentralized algorithm to solve the constrained composite optimization problem over networks in the next section.

## 3. Algorithm Development

The introduction with respect to the design process of the proposed algorithm is provided in this section.

Notice that Problem (5) is an unconstrained problem. According to [32] (Proposition 19.20), we obtain the following Lagrangian function:

(6) $$\begin{array}{c} \begin{array}{c} L=\sum_{i=1}^{m}\left(f_{i}\left(x_{i}\right)+g_{i}\left(x_{i}\right)+v_{i}^{T}x_{i}-\delta_{A_{i}}^{*}\left(v_{i}\right)+u_{i}^{T}x_{i}-\delta_{\Omega_{i}}^{*}\left(u_{i}\right)\right)\\ \;\;\;+\sum_{i=1}^{m}\sum_{\left(i,j\right)\in E}\left(w_{\left(i,j\right)}^{T}N_{\left(i,j\right)}x-\delta_{C_{\left(i,j\right)}}^{*}\left(w_{\left(i,j\right)}\right)\right), \end{array} \end{array}$$

where $v_{i}\in R^{n}$, $u_{i}\in R^{n}$ and $w_{\left(i,j\right)}\in R^{2n}$ are dual variables, and $\delta_{A_{i}}^{*}$, $\delta_{\Omega_{i}}^{*}$ and $\delta_{C_{\left(i,j\right)}}^{*}$ are the conjugate functions of $\delta_{A_{i}}$, $\delta_{\Omega_{i}}$, $\delta_{C_{\left(i,j\right)}}$, respectively. Notice that $w_{\left(i,j\right)}={\left(w_{ij}^{⊤},w_{ji}^{⊤}\right)}^{⊤}\in R^{2n}$ is an edge-based variable, where $w_{ij}\in R^{n}$ is the local variable of agent *i* and $w_{ji}\in R^{n}$ is for agent *j*. Then, the last term of the Lagrangian function (6) satisfies:

$$\begin{array}{c} \sum_{i=1}^{m}\sum_{\left(i,j\right)\in E}\left(w_{\left(i,j\right)}^{T}N_{\left(i,j\right)}x-\delta_{C_{\left(i,j\right)}}^{*}\left(w_{\left(i,j\right)}\right)\right)=\sum_{i=1}^{m}\sum_{j\in N_{i}}\left(w_{\left(i,j\right)}^{T}N_{\left(i,j\right)}x-\delta_{C_{\left(i,j\right)}}^{*}\left(w_{\left(i,j\right)}\right)\right), \end{array}$$

Thus, the Lagrangian function (6) can also be written as

(7) $$\begin{array}{c} \begin{array}{c} L=\sum_{i=1}^{m}\left(f_{i}\left(x_{i}\right)+g_{i}\left(x_{i}\right)+v_{i}^{T}x_{i}-\delta_{A_{i}}^{*}\left(v_{i}\right)+u_{i}^{T}x_{i}-\delta_{\Omega_{i}}^{*}\left(u_{i}\right)\right)\\ \;\;+\sum_{i=1}^{m}\sum_{j\in N_{i}}\left(w_{\left(i,j\right)}^{T}N_{\left(i,j\right)}x-\delta_{C_{\left(i,j\right)}}^{*}\left(w_{\left(i,j\right)}\right)\right), \end{array} \end{array}$$

Taking the partial derivatives of the Lagrangian function (7) and combining the operator splitting method [29], we propose a new update flow as follows:   

(8) $$\begin{array}{c} \left\{\begin{array}{c} {\bar{w}}_{\left(i,j\right)}^{k}={\mathrm{prox}}_{\omega_{\left(i,j\right)}\delta_{C_{\left(i,j\right)}}^{*}}\left(w_{\left(i,j\right)}^{k}+\omega_{\left(i,j\right)}\left(N_{\left(i,j\right)}x^{k}\right)\right),\\ {\bar{u}}_{i}^{k}={\mathrm{prox}}_{\mu_{i}\delta_{\Omega_{i}}^{*}}\left(u_{i}^{k}+\mu_{i}x_{i}^{k}\right),\\ {\bar{v}}_{i}^{k}={\mathrm{prox}}_{\sigma_{i}\delta_{A_{i}}^{*}}\left(v_{i}^{k}+\sigma_{i}x_{i}^{k}\right),\\ x_{i}^{k+1}={\mathrm{prox}}_{\gamma_{i}g_{i}}\left(\begin{array}{c} x_{i}^{k}-\gamma_{i}\nabla f_{i}\left(x_{i}^{k}\right)-\gamma_{i}{\bar{v}}_{i}^{k}-\gamma_{i}{\bar{u}}_{i}^{k}-\gamma_{i}\sum_{j\in N_{i}}C_{ij}^{⊤}{\bar{w}}_{ij}^{k} \end{array}\right),\\ w_{\left(i,j\right)}^{k+1}={\bar{w}}_{\left(i,j\right)}^{k}+\omega_{\left(i,j\right)}\left(N_{\left(i,j\right)}x^{k+1}-N_{\left(i,j\right)}x^{k}\right),\\ u_{i}^{k+1}={\bar{u}}_{i}^{k}+\mu_{i}\left(x_{i}^{k+1}-x_{i}^{k}\right),\\ v_{i}^{k+1}={\bar{v}}_{i}^{k}+\sigma_{i}\left(x_{i}^{k+1}-x_{i}^{k}\right), \end{array}\right. \end{array}$$

where ${\bar{w}}_{\left(i,j\right)}={\left({\bar{w}}_{\left(i,j\right)}^{⊤},{\bar{w}}_{\left(j,i\right)}^{⊤}\right)}^{⊤}\in R^{2n}$, ${\bar{u}}_{i}\in R^{n}$, ${\bar{v}}_{i}\in R^{n}$ are the auxiliary variables, and $\gamma_{i}$, $\sigma_{i}$, and $\mu_{i}$ are positive stepsizes. Notice that the stepsizes are uncoordinated, which can be selected independently related to different agents and enjoy their own acceptable ranges. Additionally, the edge-based parameters $\omega_{\left(i,j\right)}$ can be seen as inherent parameters of the communication network, revealing the quality of the communication.

The steps related to the edge-based variables in update flow (8) cannot be conducted directly, so we next replace them with the agent-based variables. We apply the Moreau decomposition to the first step in update flow (8) such that for the second term on the right side, we have

(9) $$\begin{array}{c} {\mathrm{prox}}_{\omega_{\left(i,j\right)}^{-1}\delta_{C_{\left(i,j\right)}}}\left(\omega_{\left(i,j\right)}^{-1}w_{\left(i,j\right)}^{k}+N_{\left(i,j\right)}x^{k}\right)=\underset{y\in C_{\left(i,j\right)}}{\arg\min}\left\{∥y-\left(\omega_{\left(i,j\right)}^{-1}w_{\left(i,j\right)}^{k}+N_{\left(i,j\right)}x^{k}\right)∥\right\}. \end{array}$$

Define (9) as the projection $P_{C_{\left(i,j\right)}}\left(\omega_{\left(i,j\right)}^{-1}w_{\left(i,j\right)}^{k}+N_{\left(i,j\right)}x^{k}\right)$. Then, according to the definition of the set $C_{\left(i,j\right)}$, the projection has the following explicit expression:

$$P_{C_{\left(i,j\right)}}\left[\begin{array}{c} a_{1}\\ a_{2} \end{array}\right]=\frac{1}{2}\left[\begin{array}{c} a_{1}-a_{2}\\ a_{2}-a_{1} \end{array}\right].$$

Thus, for i∈V, $j\in N_{i}$, the update step for ${\bar{w}}_{\left(i,j\right)}$ can be decomposed into

(10) $$\begin{array}{c} {\bar{w}}_{ij}^{k}=\frac{1}{2}\left(w_{ij}^{k}+w_{ji}^{k}\right)+\frac{\omega_{\left(i,j\right)}}{2}\left(C_{ij}x_{i}^{k}+C_{ji}x_{j}^{k}\right). \end{array}$$

Moreover, the update step for $w_{\left(i,j\right)}$ can be replaced by

(11) $$\begin{array}{c} w_{ij}^{k+1}={\bar{w}}_{ij}^{k}+\omega_{\left(i,j\right)}C_{ij}\left(x_{i}^{k+1}-x_{i}^{k}\right). \end{array}$$

Combining the update flow (8), (10) and (11), we finally propose the decentralized algorithm for Problem (1) in Algorithm 1.

Here, we directly give the stepsize condition of Algorithm 1 in the following assumption. The specific theoretical origin of this condition can be found in the convergence analysis section.

**Assumption** **2.** *(Stepsize conditions)*

*For any agent i∈V and $j\in N_{i}$, the stepsizes $\gamma_{i}$, $\mu_{i}$, $\sigma_{i}$ and $\omega_{\left(i,j\right)}$ are positive. Let the following condition hold:*

$$\gamma_{i}<\frac{1}{\frac{\beta_{i}}{2}+\mu_{i}+\sigma_{i}+\sum_{j\in N_{i}}\omega_{\left(i,j\right)}},$$

*where $\beta_{i}$ is the Lipschitz constant for the gradient $\nabla f_{i}$.*

**Algorithm 1** The Decentralized Algorithm
Initialization: For each agent i∈V and all $j\in N_{i}$, let ${\bar{w}}_{ij}^{0}\in R^{n}$, ${\bar{u}}_{i}^{0}\in R^{n}$, ${\bar{v}}_{i}^{0}\in R^{n}$, $x_{i}^{0}\in R^{n}$, $w_{ij}^{0}\in R^{n}$, $u_{i}^{0}\in R^{n}$ and $v_{i}^{0}\in R^{n}$.For k=0,1,2,⋯ doEach agent *i* repeats, for all $j\in N_{i}$,
$$\begin{array}{cc} \;\;\;\;{\bar{w}}_{ij}^{k} & =\frac{1}{2}\left(w_{ij}^{k}+w_{ji}^{k}\right)+\frac{\omega_{\left(i,j\right)}}{2}\left(C_{ij}x_{i}^{k}+C_{ji}x_{j}^{k}\right),\\ {\bar{u}}_{i}^{k} & =\mathrm{pro}x_{\mu_{i}\delta_{\Omega_{i}}^{*}}\left(u_{i}^{k}+\mu_{i}x_{i}^{k}\right),\\ {\bar{v}}_{i}^{k} & =\mathrm{pro}x_{\sigma_{i}\delta_{A_{i}}^{*}}\left(v_{i}^{k}+\sigma_{i}x_{i}^{k}\right),\\ x_{i}^{k+1} & =\mathrm{pro}x_{\tau_{i}g_{i}}\left(\begin{array}{c} x_{i}^{k}-\gamma_{i}\nabla f_{i}\left(x_{i}^{k}\right)-\gamma_{i}{\bar{u}}_{i}^{k}-\gamma_{i}{\bar{v}}_{i}^{k}-\gamma_{i}\sum_{j\in N_{i}}C_{ij}^{⊤}{\bar{w}}_{ij}^{k} \end{array}\right),\\ w_{ij}^{k+1} & ={\bar{w}}_{ij}^{k}+\omega_{\left(i,j\right)}C_{ij}\left(x_{i}^{k+1}-x_{i}^{k}\right),\\ u_{i}^{k+1} & ={\bar{u}}_{i}^{k}+\mu_{i}\left(x_{i}^{k+1}-x_{i}^{k}\right),\\ v_{i}^{k+1} & ={\bar{v}}_{i}^{k}+\sigma_{i}\left(x_{i}^{k+1}-x_{i}^{k}\right). \end{array}$$Agent *i* sends $w_{ij}^{k+1}$, ${\bar{w}}_{ij}^{k}$, $C_{ij}x_{i}^{k+1}$ to all of its neighbors.

End

Output: The sequence ${\left(x_{i}^{k}\right)}_{k=1}^{\infty }$ to estimate the optimal solution.


## 4. Convergence Analysis

In this section, we first establish the compact form with operators of the proposed algorithm. Then, the results of the theoretical analysis are provided.

For i∈V, $j\in N_{i}$, we make the following definitions. Let *w* and $\bar{w}$ represent the variables stacked by $w_{\left(i,j\right)}$ and ${\bar{w}}_{\left(i,j\right)}$, respectively. Define vectors $u=col\;\left(u_{1},u_{2},\ldots ,u_{m}\right),v=col\;\left(v_{1},v_{2},\ldots ,v_{m}\right),\bar{u}=col\;\left({\bar{u}}_{1},{\bar{u}}_{2},\ldots ,{\bar{u}}_{m}\right)$ and $\bar{v}=col\;\left({\bar{v}}_{1},{\bar{v}}_{2},\ldots ,{\bar{v}}_{m}\right)$. Then, we let $W=\mathrm{blkdiag}{\left\{\omega_{\left(i,j\right)}I_{2n}\right\}}_{(i,j)\in E}$, $\Gamma =\mathrm{blkdiag}{\left\{\gamma_{i}I_{n}\right\}}_{i\in V}$, $\Sigma =\mathrm{diag}{\left\{\sigma_{i}I_{n}\right\}}_{i\in V}$ and $M=\mathrm{blkdiag}{\left\{\mu_{i}I_{n}\right\}}_{i\in V}$ be the stepsize matrices. Then $C=\prod_{\left(i,j\right)\in E}C_{\left(i,j\right)}$, $\Omega =\prod_{i\in V}\Omega_{i}$ and $A=\prod_{i\in V}A_{i}$ hold such that there exist $\delta_{C}=\sum_{\left(i,j\right)\in E}\delta_{C_{\left(i,j\right)}}$, $\delta_{\Omega }=\sum_{i\in V}\delta_{\Omega_{i}}$ and $\delta_{A}=\sum_{i\in V}\delta_{A_{i}}$. The linear operator $N:x\mapsto {\left(N_{\left(i,j\right)}x\right)}_{\left(i,j\right)\in E}$ is stacked by $N_{\left(i,j\right)}$. Considering the resolvent of the proximal operator, the update flow (8) leads to the following equalities:

(12) $$\begin{array}{c} \left\{\begin{array}{c} \partial \delta_{C}^{*}\left({\bar{w}}^{k}\right)+W^{-1}{\bar{w}}^{k}=W^{-1}w^{k}+Nx^{k},\\ \partial \delta_{\Omega }^{*}\left({\bar{u}}^{k}\right)+M^{-1}{\bar{u}}^{k}=M^{-1}u^{k}+x^{k},\\ \partial \delta_{A}^{*}\left({\bar{v}}^{k}\right)+\Sigma^{-1}{\bar{v}}^{k}=\Sigma^{-1}v^{k}+x^{k},\\ \partial g\left({\bar{x}}^{k}\right)+\Gamma^{-1}{\bar{x}}^{k}+{\bar{u}}^{k}+{\bar{v}}^{k}+N^{⊤}{\bar{w}}^{k}=\Gamma^{-1}x^{k}-\nabla f\left(x^{k}\right),\\ w^{k+1}={\bar{w}}^{k}+WN{\bar{x}}^{k}-WNx^{k},\\ u^{k+1}={\bar{u}}^{k}+M{\bar{x}}^{k}-Mx^{k},\\ v^{k+1}={\bar{v}}^{k}+\Sigma {\bar{x}}^{k}-\Sigma x^{k}, \end{array}\right. \end{array}$$

where ${\bar{x}}^{k}=x^{k+1}$ is the auxiliary variable.

Define two variables $U=col\left(w,u,v,x\right)$ and $\bar{U}=col\left(\bar{w},\bar{u},\bar{v},\bar{x}\right)$. Based on the equalities in (12), Algorithm 1 is equivalent to the following compact form described by the operators:

(13) $$\begin{array}{c} \left\{\begin{array}{c} {\bar{U}}^{k}={\left(T_{H}+T_{A}\right)}^{-1}\left(T_{H}-T_{Q}-T_{D}\right)U^{k},\\ U^{k+1}=U^{k}+T_{S}^{-1}\left(T_{H}-T_{Q}\right)\left({\bar{U}}^{k}-U^{k}\right), \end{array}\right. \end{array}$$

where the operators are given as follows:

$$T_{H}:U\mapsto {\left(\begin{array}{c} {\left(W^{-1}w\right)}^{⊤},{\left(M^{-1}u\right)}^{⊤},{\left(\Sigma^{-1}v\right)}^{⊤},{\left(N^{⊤}w+u+v+\Gamma^{-1}x\right)}^{⊤} \end{array}\right)}^{⊤},$$

$$T_{A}:U\mapsto {\left(\partial \delta_{C}^{*}{\left(w\right)}^{⊤},\partial \delta_{\Omega }^{*}{\left(u\right)}^{⊤},\partial \delta_{A}^{*}{\left(v\right)}^{⊤},\partial g{\left(x\right)}^{⊤}\right)}^{⊤},$$

$$T_{Q}:U\mapsto {\left({\left(-Nw\right)}^{⊤},-u^{⊤},-v^{⊤},{\left(N^{⊤}w+u+v\right)}^{⊤}\right)}^{⊤},$$

$$T_{D}:U\mapsto {\left(0_{n},0_{n},0_{n},\nabla f{\left(x\right)}^{⊤}\right)}^{⊤},$$

$$T_{S}:U\mapsto {\left({\left(Ww\right)}^{⊤},{\left(Mu\right)}^{⊤},{\left(\Sigma v\right)}^{⊤},{\left(\Gamma x\right)}^{⊤}\right)}^{⊤}.$$

Consider one iteration of the proposed algorithm as an operator *T*. Then we let $U^{*}=col\left(x^{*},w^{*},\;u^{*},v^{*}\right)$ be the fixed point of the operator *T* such that $U^{*}=TU^{*}$. Next, we conduct the convergence analysis.

**Lemma** **1.** (Optimal analysis) Let Assumption 1 be satisfied. The fixed point $U^{*}$ related to the operator T meets the first-order optimal conditions of the objective problem, and $x^{*}\in S$ is an optimal solution.

**Proof.**  Substituting the fixed point into (12), we have the following set of equalities:

$$\left\{\begin{array}{c} \partial \delta_{C}^{*}\left(w^{*}\right)-Nx^{*}=0,\\ \partial \delta_{\Omega }^{*}\left(u^{*}\right)-x^{*}=0,\\ \partial \delta_{A}^{*}\left(v^{*}\right)-x^{*}=0,\\ \partial g\left(x^{*}\right)+\nabla f\left(x^{*}\right)+u^{*}+v^{*}+N^{⊤}w^{*}=0, \end{array}\right.$$

which is also the KKT condition of the Lagrangian function (6). Therefore, $x^{*}$ is an optimal solution to problem (1). □

The relationship between the fixed point and the optimal solution is ensured by Lemma 1. Split the operator $T_{H}$ as $T_{H}=T_{P}+T_{K}$, where we let

$$T_{P}=\left[\begin{array}{cccc} W^{-1} & 0 & 0 & \frac{1}{2}N\\ 0 & M^{-1} & 0 & \frac{1}{2}I\\ 0 & 0 & \Sigma^{-1} & \frac{1}{2}I\\ \frac{1}{2}N^{⊤} & \frac{1}{2}I & \frac{1}{2}I & \Gamma^{-1} \end{array}\right],$$

$$T_{K}=\left[\begin{array}{cccc} 0 & 0 & 0 & -\frac{1}{2}N\\ 0 & 0 & 0 & -\frac{1}{2}I\\ 0 & 0 & 0 & -\frac{1}{2}I\\ \frac{1}{2}N^{⊤} & \frac{1}{2}I & \frac{1}{2}I & 0 \end{array}\right],$$

and further define another linear operator

$$T_{\tilde{P}}=\left[\begin{array}{cccc} W^{-1} & 0 & 0 & -\frac{1}{2}N\\ 0 & M^{-1} & 0 & -\frac{1}{2}I\\ 0 & 0 & \Sigma^{-1} & -\frac{1}{2}I\\ -\frac{1}{2}N^{⊤} & -\frac{1}{2}I & -\frac{1}{2}I & \Gamma^{-1} \end{array}\right].$$

With these definitions above, the following lemma provides the property of the operator *T* for convergence analysis.

**Lemma** **2.** Under Assumption 1, there exists the following inequality for $U^{*}$:

$${\left({\bar{U}}^{k}-U^{*}\right)}^{⊤}T_{D}\left(U^{k}-U^{*}\right)\geq -\frac{1}{4}{∥x^{k}-{\bar{x}}^{k}∥}_{B}^{2},$$

*where $B=\mathrm{blkdiag}\left\{\beta_{i}I_{n}\right\}$ for i∈V is the Lipschitz parameter matrix.*

**Proof.**  With the definition of operator $T_{D}$, we have the equality

(14) $$\begin{array}{cc} \; & {\left({\bar{U}}^{k}-U^{*}\right)}^{⊤}T_{D}\left(U^{k}-U^{*}\right)\\ \; & \;=-{\left(x^{k}-{\bar{x}}^{k}\right)}^{⊤}\left(\nabla f\left(x^{k}\right)-\nabla f\left(x^{*}\right)\right)+{\left(x^{k}-x^{*}\right)}^{⊤}\left(\nabla f\left(x^{k}\right)-\nabla f\left(x^{*}\right)\right). \end{array}$$

According to [32] (Theorem 18.16), for i∈V, $\nabla f_{i}$ is cocoercive, i.e., it holds

(15) $$\begin{array}{c} {∥\nabla f\left(x^{k}\right)\;-\;\nabla f\left(x^{*}\right)∥}_{B^{-1}}^{2}\leq {\left(x^{k}-x^{*}\right)}^{⊤}\left(\nabla f\left(x^{k}\right)\;-\;\nabla f\left(x^{*}\right)\right)\;. \end{array}$$

Note that for any vector *a* and *b* in the same dimension and a diagonal positive definite matrix *V*, then there exists the inequality $x^{⊤}y\leq {∥x∥}_{V}^{2}+\frac{1}{4}{∥y∥}_{V^{-1}}^{2}$. Hence, we have

(16) $$\begin{array}{c} {\left(x^{k}-{\bar{x}}^{k}\right)}^{⊤}\left(\nabla f\left(x^{k}\right)-\nabla f\left(x^{*}\right)\right)\leq {∥\nabla f\left(x^{k}\right)-\nabla f\left(x^{*}\right)∥}_{B^{-1}}^{2}+\frac{1}{4}{∥x_{k}-{\bar{x}}_{k}∥}_{B}^{2}. \end{array}$$

Combining (14)–(16), we can obtain the objective inequality and end the proof. □

**Lemma** **3.** Under Assumption 1, there exists the following inequality for $U^{*}$:

(17) $$\begin{array}{c} {∥U^{k+1}-U^{*}∥}_{T_{S}}^{2}-{∥U^{k}-U^{*}∥}_{T_{S}}^{2}\leq {∥U^{k+1}-U^{k}∥}_{T_{S}-2T_{\tilde{P}}}^{2}+\frac{1}{2}{∥x^{k}-{\bar{x}}^{k}∥}_{B}^{2}, \end{array}$$

*where $T_{\tilde{P}}$ is defined before Lemma 2.*

**Proof.**  Considering the change of the optimal residual before and after one iteration, we have

(18) $$\begin{array}{cc} \; & {∥U^{k+1}-U^{*}∥}_{T_{S}}^{2}-{∥U^{k}-U^{*}∥}_{T_{S}}^{2}\\ \; & \;=-\begin{array}{c} {∥U^{k+1}-U^{k}∥}_{T_{S}}^{2}+2{\left(U^{k+1}-U^{*}\right)}^{⊤}T_{S}\left(U^{k+1}-U^{k}\right) \end{array}\\ \; & \;=\begin{array}{c} {∥U^{k+1}-U^{k}∥}_{T_{S}}^{2}+2{\left(U^{k}-U^{*}\right)}^{⊤}T_{S}\left(U^{k+1}-U^{k}\right) \end{array}. \end{array}$$

From the second step of the update flow (13), there exists

$$T_{S}\left(U^{k+1}-U^{k}\right)=\left(T_{H}-T_{Q}\right)\left({\bar{U}}^{k}-U^{k}\right),$$

such that the equality (18) leads to

$$\begin{array}{cc} \; & {∥U^{k+1}-U^{*}∥}_{T_{S}}^{2}-{∥U^{k}-U^{*}∥}_{T_{S}}^{2}\\ \; & \;={∥U^{k+1}-U^{k}∥}_{T_{S}}^{2}\\ \; & \;\;+2{\left({\bar{U}}^{k}-U^{*}\right)}^{⊤}\left(T_{H}-T_{Q}\right)\left({\bar{U}}^{k}-U^{k}\right)\\ \; & \;\;-2{\left({\bar{U}}^{k}-U^{k}\right)}^{⊤}\left(T_{H}-T_{Q}\right)\left({\bar{U}}^{k}-U^{k}\right)\\ \; & \;\leq {∥U^{k+1}-U^{k}∥}_{T_{S}}^{2}\\ \; & \;\;+2{\left({\bar{U}}^{k}-U^{*}\right)}^{⊤}\left(T_{H}-T_{Q}\right)\left({\bar{U}}^{k}-U^{k}\right)\\ \; & \;\;-2{\left({\bar{U}}^{k}-U^{k}\right)}^{⊤}T_{P}\left({\bar{U}}^{k}-U^{k}\right). \end{array}$$

From the first step of the update flow (13), it holds that

$$\left(T_{H}-T_{Q}\right)\left({\bar{U}}^{k}-U^{k}\right)=-\left(T_{D}U^{k}+T_{Q}{\bar{U}}^{k}+T_{A}{\bar{U}}^{k}\right).$$

Thus, we further have

(19) $$\begin{array}{cc} \; & {∥U^{k+1}-U^{*}∥}_{T_{S}}^{2}-{∥U^{k}-U^{*}∥}_{T_{S}}^{2}\\ \; & \;\leq {∥U^{k+1}-U^{k}∥}_{T_{S}}^{2}-2{\left({\bar{U}}^{k}-U^{k}\right)}^{⊤}T_{P}\left({\bar{U}}^{k}-U^{k}\right)\\ \; & \;\;-2{\left({\bar{U}}^{k}-U^{*}\right)}^{⊤}\left(T_{D}U^{k}+T_{Q}{\bar{U}}^{k}+T_{A}U^{*}\right)\\ \; & \;\;-2{\left({\bar{U}}^{k}-U^{*}\right)}^{⊤}\left(T_{A}{\bar{U}}^{k}-T_{A}U^{*}\right). \end{array}$$

Then, we discuss the right side of (19). Note that Lemma 1 proves the equivalence between the fixed point and the optimal solution. Substituting the property of fixed points into the update flow (13), we obtain $U^{*}={\bar{U}}^{*}$ and

$$T_{A}U^{*}=-T_{Q}U^{*}-T_{D}U^{*}.$$

Hence, the third term on the right side of (19) satisfies

(20) $$\begin{array}{cc} \; & {\left({\bar{U}}^{k}-U^{*}\right)}^{⊤}\left(T_{D}U^{k}+T_{Q}{\bar{U}}^{k}+T_{A}U^{*}\right)\\ \; & \;={\left({\bar{U}}^{k}-U^{*}\right)}^{⊤}T_{D}\left(U^{k}-U^{*}\right)+{\left({\bar{U}}^{k}-U^{*}\right)}^{⊤}T_{Q}\left({\bar{U}}^{k}-U^{*}\right)\\ \; & \;\geq -\frac{1}{4}{∥x^{k}-{\bar{x}}^{k}∥}_{B}^{2}+{\left({\bar{U}}^{k}-U^{*}\right)}^{⊤}T_{Q}\left({\bar{U}}^{k}-U^{*}\right), \end{array}$$

where the inequality is based on Lemma 2. Notice that the operator $T_{A}$ is monotone [32] (Theorem 21.2 and Proposition 20.23), i.e., it holds

(21) $$\begin{array}{c} {\left({\bar{U}}^{k}-U^{*}\right)}^{⊤}\left(T_{A}{\bar{U}}^{k}-T_{A}U^{*}\right)\geq 0. \end{array}$$

Since the linear operator $T_{Q}$ is a skew-symmetric matrix, it is monotone [29]. Combining (19)–(21), we obtain

(22) $$\begin{array}{cc} \; & {∥U^{k+1}-U^{*}∥}_{T_{S}}^{2}-{∥U^{k}-U^{*}∥}_{T_{S}}^{2}\\ \; & \;\leq {∥U^{k+1}-U^{k}∥}_{T_{S}}^{2}-2{\left({\bar{U}}^{k}-U^{k}\right)}^{⊤}T_{P}\left({\bar{U}}^{k}-U^{k}\right)+\frac{1}{2}{∥x^{k}-{\bar{x}}^{k}∥}_{B}^{2}. \end{array}$$

From the second step of the update flow (13), it holds

$${\bar{U}}^{k}-U^{k}={\left(T_{H}-T_{Q}\right)}^{-1}T_{S}\left(U^{k+1}-U^{k}\right),$$

where $T_{H}$, $T_{Q}$ and $T_{S}$ are the linear operators. Considering that $T_{P}$ is also a linear operator, the second term on the right side of (22) has an equivalent form:

(23) $$\begin{array}{c} {\left({\bar{U}}^{k}\;-\;U^{k}\right)}^{⊤}\;T_{P}\left({\bar{U}}^{k}\;-\;U^{k}\right)={\left(U^{k+1}\;-\;U^{k}\right)}^{⊤}\;T_{\tilde{P}}\left(U^{k+1}\;-\;U^{k}\right). \end{array}$$

Substituting (23) into (22), we complete the proof. □

Summarizing the above lemmas, the following theorem supplies the convergence results.

**Theorem** **1.** *When Assumption 1 and 2 are satisfied, for the sequence ${\left(U^{k}\right)}_{k\geq 0}$ generated by the operator T, we have*

(24) $$\begin{array}{c} {∥U^{k+1}-U^{*}∥}_{T_{s}}^{2}-{∥U^{k}-U^{*}∥}_{T_{s}}^{2}\leq -{∥U^{k+1}-U^{k}∥}_{2\left(T_{\tilde{P}}-\tilde{B}\right)-T_{S}}^{2}, \end{array}$$

where $\tilde{B}=\mathrm{blkdiag}\left\{0_{n\times n},0_{n\times n},0_{n\times n},\frac{1}{4}B\right\}$. Then, the sequence ${\left(U^{k}\right)}_{k\geq 0}$ has sublinear rate $O\left(1/k\right)$, and the sequence ${\left(x^{k}\right)}_{k\geq 0}$ converges to an optimal solution $x^{*}\in S$.

**Proof.**  With the definition of $\tilde{B}$, we have the following equality:

(25) $$\begin{array}{c} \frac{1}{4}{∥x^{k}-{\bar{x}}^{k}∥}_{B}^{2}={∥U^{k+1}-U^{k}∥}_{\tilde{B}}^{2}. \end{array}$$

Substituting (25) into (17), we obtain the inequality (24). Note that under Assumption 2, the matrix $2\left(T_{\tilde{P}}-\tilde{B}\right)-T_{S}$ is positive definite. Hence, the sequence ${\left(U^{k}\right)}_{k\geq 0}$ converges to the fixed point $U^{*}$. Meanwhile, utilizing [10] (Proposition 1) results in the $O\left(1/k\right)$ rate, and based on Lemma 1, the convergence of ${\left(x^{k}\right)}_{k\geq 0}$ holds. □

In Theorem 1, the positive definite property is needed for the induced matrices, which leads to the stepsize conditions in Assumption 2.

## 5. Numerical Simulation

The correctness of the theoretical analysis is verified through numerical simulation on a constrained optimization problem over networks in this section.

The constrained quadratic programming problem [33] is considered in the experiments, which has the formulation as follows:

(26a) $$\begin{array}{cc} \; & \min_{\tilde{x}}\;\;\;\sum_{i=1}^{m}{∥\tilde{x}∥}_{E_{i}}^{2}+e_{i}^{⊤}\tilde{x}+\rho_{i}{∥\tilde{x}∥}_{1}, \end{array}$$

(26b) $$\begin{array}{cc} \; & \;s.t.\;\;\;A_{i}\tilde{x}=b_{i},\;\;\;\;i\in V, \end{array}$$

(26c) $$\begin{array}{cc} \; & \;\;\;\;\;\;\;\;\;\;{\tilde{x}}^{\min}\leq \tilde{x}\leq {\tilde{x}}^{\max},\;i\in V, \end{array}$$

where matrix $E_{i}\in R^{n\times n}$ is diagonal and positive definite, $e_{i}\in R^{n}$ is a vector, and $\rho_{i}$ is the penalty factor. Both $x_{i}^{\min}$ and $x_{i}^{\max}$ are vectors with constants, which give the bounds of the decision variable $\tilde{x}$. In the light of (1), we can set $f_{i}\left(\tilde{x}\right)={∥\tilde{x}∥}_{E_{i}}^{2}+e_{i}^{T}\tilde{x}$ and $g_{i}\left(\tilde{x}\right)=\rho_{1}{∥\tilde{x}∥}_{1}$.

**Figure 1:**
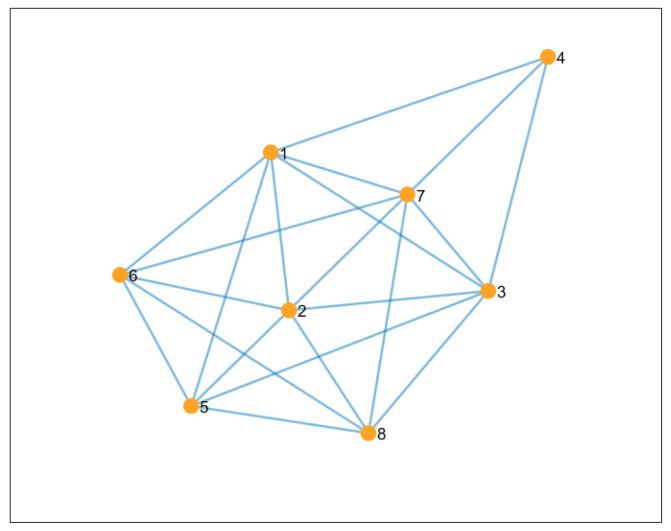
The 8-agents communication network.

In this case, the dimension of the decision variable is set as n=4, and we let r=1. For i∈V, the paramount data of Problem (26) are selected randomly. The elements of matrix $E_{i}$ are in [1,2], and the elements of the linear operator $A_{i}$ are in [1,15]. Both vectors $e_{i}$ and $b_{i}$ take values in [−5,5]. The box constraints are considered as [−2.5,2.5]. Then, we set the uncoordinated setpsizes randomly as $\gamma_{i}\in \left[0.005,0.006\right]$, while $\sigma_{i}$, $\mu_{i}$ and $\omega_{\left(i,j\right)}$ are in [5,6]. The numerical experiments are performed over the generated network with eight agents, which is displayed in Figure 1. The simulations are carried by running the distributed algorithms on a laptop with Intel(R) Core i5-5500U CPU @ 2.40 GHz, 8.0 GB of RAM, and Matlab R2016a on Windows 10 operating system.

**Figure 2:**
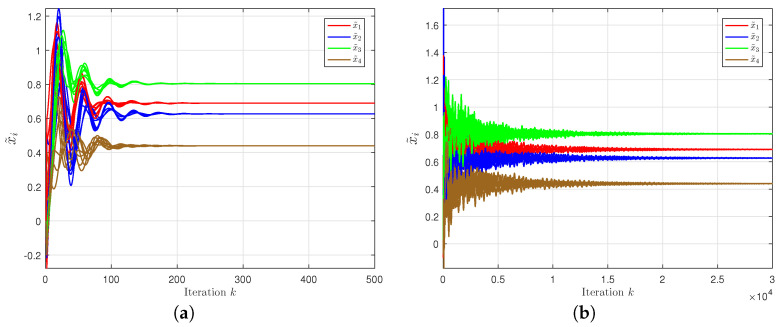
Trajectory of variable $x_{i}^{k}$. (**a**) Proposed algorithm. (**b**) Node-based consensus algorithm.

**Figure 3:**
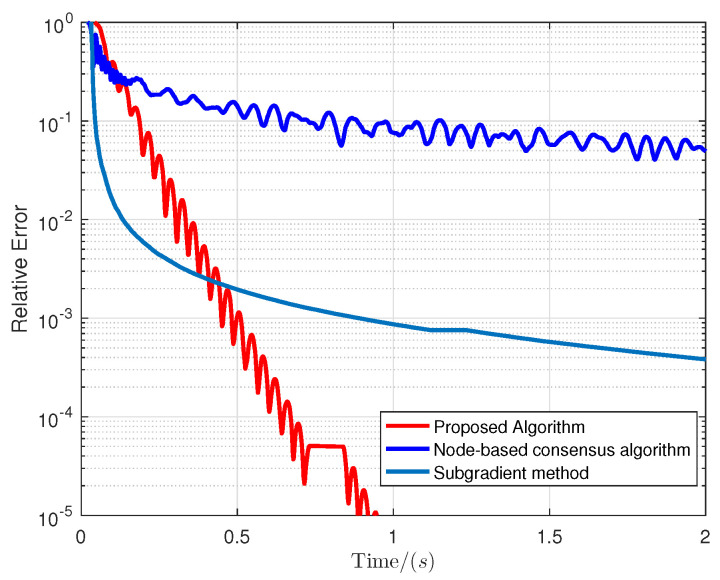
Convergence performance comparison.

The simulation results are shown in Figure 2 and Figure 3. The transient behaviors of each component of $x_{i}^{k}$ is displayed in Figure 2, in which a node-based consensus algorithm [34] is introduced as a comparative profile. Note that the obtained optimal solution from the proposed algorithm is in line with that of the node-based consensus one, i.e., $x^{*}={\left[0.6900,0.6270,\;0.8046,\;0.4400\right]}^{T}$, but the latter achieves a stable consensus after 15,000 iterations. Figure 3 shows that our proposed algorithm outperforms the node-based and subgradient algorithms [35] in terms of convergence performance by evaluating the relative errors $\sum_{i=1}^{m}∥x_{i}^{k}-{\tilde{x}}^{*}∥/m∥{\tilde{x}}^{*}∥$.

## 6. Conclusions

In this paper, a distributed algorithm based on proximal operators has been designed to deal with discussed a class of distributed composite optimization problems, in which the local function has a smooth and nonsmooth structure and the decision variable abides by both affine and feasible constraints. Distinguishing attributes of the proposed algorithm include the use of uncoordinated stepsizes and the edge-based communication that avoids the dependency on Laplacian weight matrices. Meanwhile, the algorithm has been verified in theory and simulation. However, there are still some aspects worthy of improvement in this paper. For example, it is worth adopting efficient accelerated protocols (such as the Nesterov-based method and heavy ball method) to improve the convergence rate and developing asynchronous distributed algorithms to deal with the issue of communication latency. In addition, more general optimization models and more efficient algorithms should be investigated in order to address potential applications, e.g., [36,37,38] with nonconvex objectives, coupled and nonlinear constraints.

## Data Availability

Not applicable.
